# Immunostimulatory siRNA with a uridine bulge leads to potent inhibition of HBV and activation of innate immunity

**DOI:** 10.1186/s12985-021-01509-z

**Published:** 2021-02-18

**Authors:** Tingyu Lan, Zhiqiang Wei, Yulin He, Song Wan, Li Liu, Bin Cheng, Ruimin Li, Hongxia Chen, Guohua Liu, Zhongji Meng

**Affiliations:** 1 Postgraduate Training Basement of Jinzhou Medicical University, Taihe Hospital, Hubei University of Medicine, Shiyan, China; 2 Institute of Biomedical Research, Taihe Hospital, Hubei University of Medicine, No. 32, South Renmin Road, Shiyan, 442000 Hubei Province China; 3 Department of Infectious Diseases, Taihe Hospital, Hubei University of Medicine, Shiyan, China; 4 Hubei Clinical Research Center for Precise Diagnosis and Treatment of Liver Cancer, Taihe Hospital, Hubei University of Medicine, Shiyan, 442000 China; 5 Hubei Key Laboratory of Embryonic Stem Cell Research, Taihe Hospital, Hubei University of Medicine, Shiyan, 442000 China

**Keywords:** Hepatitis B virus, RNAi, microRNA-like siRNA, msiRNA, Immunostimulation, Innate immunity

## Abstract

**Background:**

Hepatitis B virus (HBV) infection is difficult to cure. HBV-specific immune tolerance plays a key role in HBV persistence, and enhancing cellular and humoral immunity will improve the control of HBV infection. The purpose of the study was to explore the anti-HBV and immunostimulatory effects of msiRNAs that introduce unpaired uridine bulges in the passenger strand.

**Methods:**

msiRNAs targeting the HBV S and X genes were designed and named msiHBs and msiHBx, respectively. HepG2 cells were cotransfected with siRNA or msiRNA and the HBV replication-competent plasmid pHY106-wta or pHY106-X15. HepG2.215 cells were transfected with siRNA or msiRNA. The levels of HBsAg, HBeAg, and the cytokines TNF-α, IFN-α, IFN-β, IL-1α, and IL-6 in the culture supernatant was detected by ELISA. The levels of intracellular HBV RNA, nuclear HBV replication intermediates, and HBV DNA in the supernatant were measured by quantitative RT-PCR and PCR. The levels of HBV replication intermediates were detected by Southern blotting. Peripheral blood mononuclear cells were transfected with siRNA or msiRNA, and the levels of secreted cytokines IFN-α and IFN-β were detected by ELISA. The bioactivity of type I interferons in the supernatants was detected by the virus protection assay.

**Results:**

msiHBx treatment led to a significant decrease in HBsAg (to a negative level) and HBV DNA (95.5%) in the supernatant and intrahepatocellular HBV replication intermediates (89.8%) in HepG2 cells with transient HBV replication and in HepG2.2.15 cells. There was no significant difference between msiHBx and siHBx in terms of the reduction in HBV proteins and HBV replication (P > 0.05). Compared with siHBx, msiHBx treatment of HepG2 cells transfected with the HBV replication-competent plasmid led to a significant increase in the levels of the antiviral cytokines TNF-α (3.3-fold), IFN-α (1.4-fold), and IFN-β (2.5-fold) (P < 0.01), without upregulation of the proinflammatory cytokines IL-1α and IL-6. The virus protection assay results showed msiHBx-mediated type I interferons effectively protected L929 cells against ECMV infection.

**Conclusions:**

msiHBx could effectively inhibit HBV expression and replication and induce an antiviral innate immune response without proinflammatory activation. The dual RNAi and immunostimulatory activity of msiRNAs may play an important role in the control of HBV infection.

## Background

Chronic hepatitis B (CHB), a disease caused by hepatitis B virus (HBV), is a global public health problem and the main cause of liver failure, liver cirrhosis, and hepatocellular carcinoma [1]. Currently, pegylated interferon-α (PEG-IFN-α) and nucleos(t)ide analogs (NAs) are the main drug therapies for CHB. PEG-IFN-α is effective only in a small number of CHB patients and is limited by its high cost and severe adverse effects. NAs, especially entecavir, tenofovir, and tenofovir alafenamide fumarate (TAF), are convenient to take orally and are widely used in the treatment of CHB; however, NAs cannot completely eliminate HBV and need long-term application, which may cause problems such as drug resistance [2]. Thus, it is urgent to develop more effective agents to pursue a cure for HBV infection.

It is generally accepted that high levels of HBV viral loads, hepatitis B surface antigen (HBsAg) and hepatitis B e antigen (HBeAg) lead to HBV-specific immune tolerance in patients with chronic HBV infection [3]. Persistent HBV infection causes the dysfunction and exhaustion of T cells and the downregulation and dysfunction of innate immunity-related factors/pathways, especially the Toll-like receptor (TLR) pathway. TLRs are widely expressed in immune cells, hepatocytes, and nonparenchymal liver cells (NPCs) [4, 5]. The activation of TLRs in the liver is essential for the establishment of antiviral homeostasis. The reduction in TLR expression caused by chronic HBV infection results in HBV escape from immune cell surveillance, thereby maintaining HBV-specific immune tolerance [6]. Reducing circulating and intrahepatic HBV particles and proteins is a prerequisite for rebuilding/restoring an effective HBV-specific immune response [3].

RNA interference (RNAi) technology can specifically degrade target RNA and has shown potential curative effects in anti-HBV research by significantly reducing HBsAg levels and promoting the elimination of HBsAg. A variety of small interfering RNA (siRNA)-based drugs targeting HBV sequences are currently undergoing assessment in phase I and II clinical trials and have shown promising prospects [7–9]. RNAi is an evolutionarily conserved antiviral mechanism that relies on siRNA of 21–23 base-pair-long double-stranded RNA. Interestingly, previous studies reported that some siRNAs can stimulate the innate immune response in a sequence-specific manner through the activation of retinoic acid-inducible gene-I (RIG-I)/melanoma differentiation-associated protein 5 (MDA5), protein kinase receptor (PKR), and the TLR-3 and TLR-7/8 signaling pathways [10–12]. These siRNAs are called immunostimulatory siRNAs (isiRNAs) which can degrade their target RNA to knock down the expression of the target protein on the one hand and enhance the innate immune response on the other hand. The dual function of isiRNAs may play an important role in antiviral and antitumor therapy [13].

Various isiRNAs have been tested in anti-HBV research [14, 15]. In HBV transgenic mice, tail injection of guanidinopropyl (GP)-modified siRNA resulted in a decrease in HBsAg of approximately 80% that lasted for a long time (2 weeks). Toxicity or induction of the proinflammatory factors consisting of interleukin (IL)-6, IFN-γ, and tumor necrosis factor (TNF)-α was not observed after injection of the GP-modified siRNAs [16]. The addition of a 5′-triphosphate (3p) to the siRNAs generated ligands for RIG-I. A 5′-end triphosphate siRNA (3p-siRNA) could induce a RIG-I-dependent antiviral type I IFN response in HBV-infected primary human hepatocytes and HepG2.2.15 cells. In addition, 3p-siRNAs showed more pronounced and long-term suppression of HBV DNA replication and mRNA transcription than normal siRNAs targeting the same sequences [17].

This study introduced a microRNA (miRNA)-like unpaired uridine bulge in the passenger strand of siRNAs to design novel miRNA-like siRNAs (msiRNAs) targeting the HBV sequence and explored its role in the inhibition of HBV gene expression and replication and the activation of innate immune responses.

## Methods

### siRNAs and plasmids

**Fig. 1:**
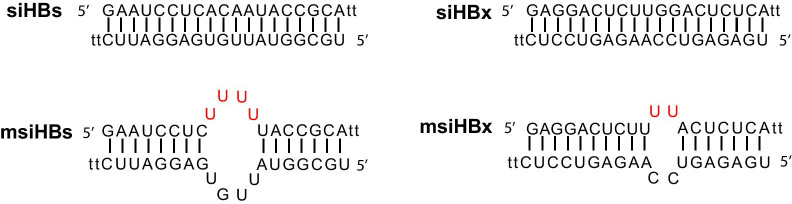
The sequences and structures of siRNAs and msiRNAs with unpaired uracil bulges. The uracil substitution at the 9–12th nucleotides of the passenger strand of siRNAs is indicated in red. siHBs, siRNA targeting the HBV S gene. siHBx, siRNA targeting the HBV X gene. msiHBs, msiRNA targeting the HBV S gene. msiHBx, msiRNA targeting the HBV X gene. siNC was purchased from Sangon Biotech (Shanghai, China) and used as a negative control

The sequences of siHBs and siHBx were obtained from a previous study [18]. msiRNAs were generated by substituting the 9-12th nucleotides of the passenger strand of siRNAs with unpaired uracil nucleotides (Fig. 1). msiRNAs targeting the HBV S and X genes with the same target sequences as siHBs and siHBx were designed and named msiHBs and msiHBx, respectively. siNC was purchased from Sangon Biotech (Shanghai, China) and used as a negative control. All siRNAs were synthesized by Sangon Biotech (Shanghai, China). The HBV replication-competent plasmid pHY106-wta (1.3 copies of HBV generated from pSM2 [19]) was a kind gift from Prof. Mengji Lu at Essen University Hospital, Germany. The HBV replication-competent plasmid pHY106-X15 was constructed by cloning a clinically derived single copy of the HBV sequence (genotype C, GenBank accession No. KM213037) into the pHY106 backbone.

### Cell culture and transfection

HepG2.2.15 cells, HepG2 cells, and Huh7 cells were cultured at 37 °C in a CO_2_ incubator. HepG2.2.15 cells were routinely cultured in RPMI-1640 medium (Gibco, America) supplemented with 10% inactivated fetal bovine serum (FBS), 100 U/mL penicillin, 100 μg/mL streptomycin (Gibco, America), 1% nonessential amino acids (NEAA), 1% HEPES, and 500 μg/mL G418 (Gibco, America). HepG2 cells and Huh7 cells were cultured in DMEM medium, supplemented with 10% inactivated FBS, 100 U/mL penicillin, 100 μg/mL streptomycin (Gibco, America), 1% NEAA, and 1% HEPES. The transfection of siRNAs and plasmids was performed with Lipofectamine 2000 transfection reagent (Invitrogen, America) according to the manufacturer’s instructions. Peripheral blood mononuclear cells (PBMCs) from CHB patients (obtained with signed informed consent) were separated by Ficoll density gradient centrifugation and cultured in RPMI-1640 + L-glutamine medium (Gibco, America) supplemented with 10% inactivated FBS. DOTAP liposomal transfection reagent (Sigma-Aldrich) was used in the transfection of PBMCs with siRNAs.

### PCR and reverse transcription (RT) PCR assays

Total DNA in the supernatant was extracted with a TIAN amp Virus DNA/RNA Kit (Tiangen, China), and 2 μl of the extracted DNA solution was subjected to real-time PCR to quantify extracellular HBV virions. HBV replication intermediates in cells were extracted according to a previous study [20], and 2 μl of the extracted DNA solution was subjected to real-time PCR. Real-time PCR was carried out by using TB Green Premix Ex Taq II (TaKaRa) in a Step-OnePlus Real-Time PCR System (Thermo Fisher Scientific) using the primers 5′-GTTGCCCGTTTGTCCTCTAATTC and 5′-GGAGGGATACATAGAGGTTCCTT. PCR was performed with the following parameters for 40 cycles: 95 °C for 5 s and 60 °C for 30 s.

Total RNA was purified from cells using TRIzol reagent (Invitrogen) according to the manufacturer’s instructions. One-step real-time RT-PCR was performed with 100 ng of total RNA using a One-step RT-PCR kit (TaKaRa) on a Step-One Plus Real-Time PCR System (Thermo Fisher Scientific) using the primers 5′-CCGTCTGTGCCTTCTCATCT and 5′-TAATCTCCTCCCCCAACTCC to detect HBV RNA levels. RT-PCR was performed with the following parameters for 40 cycles: 95 °C for 30 s, 55 °C for 30 s, and 72 °C for 30 s.

### ELISA

HBsAg and HBeAg in the culture supernatants were detected by the HBsAg ELISA Kit and HBeAg ELISA Kit (Kehua Bio engineering, China). The levels of IL-1α, IL-6, IFN-α, IFN-β, and TNF-α in the culture supernatants were quantified by ELISA kits (BD Biosciences).

### Southern blotting

The intracellular HBV replication intermediates extracted above were detected by Southern blotting using DIG high prime DNA labeling and detection starter kit II (Roche).

### Virus protection assay

The bioactivity of type I IFNs in supernatants was analyzed by the virus protection assay as described elsewhere [21]. Briefly, L929 cells were seeded into 96-well plates and cultured in 100 µl RPMI-1640 medium supplemented with 10% FBS at 37 °C and 5% CO_2_ until they reached 100% confluence. After the culture medium was discarded, 100 µl of RPMI-1640 medium with 1:2 to 1:256 dilutions of the supernatants of siRNA-treated HepG2 cells was added to the L929 cells for an additional incubation of 24 h. Then, murine encephalomyocarditis virus (EMCV) was added to the cells and incubated for another 24 h. The cells were stained and fixed with 0.1% crystal violet in 20% ethanol. A unit of IFN was defined by its ability to protect 50% of the cells from cell death.

### Statistical analyses

The results of measurement data are expressed as the means ± SEM. Statistical analyses were performed using GraphPad Prism software version 7 (La Jolla, CA, USA). Differences between groups were assessed with two-tailed Student’s t test of variance (ANOVA). P < 0.05 was considered statistically significant. All experiments were repeated independently at least three times.

## Results

### msiRNA (msiHBx) with unpaired uracil bulges showed similar effects as standard siRNA (siHBx) in knocking down HBsAg

**Fig. 2:**
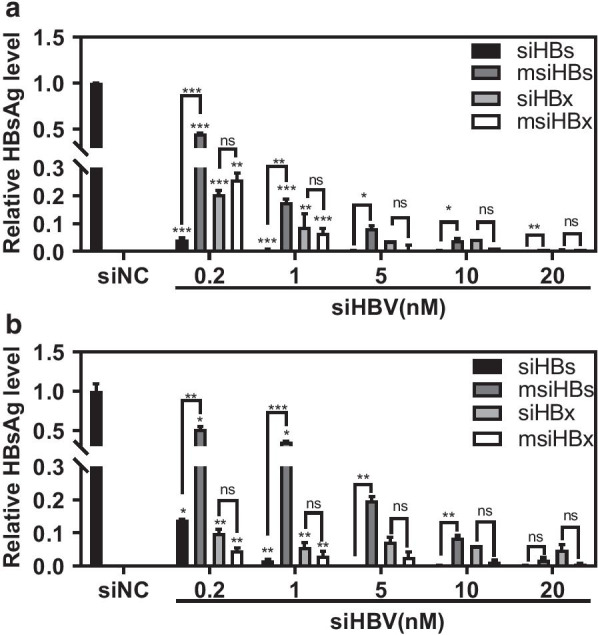
Comparison of siRNAs and msiRNAs in their ability to knock down HBsAg expression. HepG2 cells were cotransfected with 1 μg of the HBV replication-competent plasmid pHY106-wta (**a**) or pHY106-X15 (**b**) and msiHBs, msiHBx, siHBs, or siHBx at various concentrations or with 20 nM siNC as a negative control. The levels of HBsAg in supernatants were detected by ELISA at 72 h after transfection. ns, no significant difference. *p < 0.05. **p < 0.01. ***p < 0.001. siNC was purchased from Sangon Biotech (Shanghai, China) and used as a negative control. siHBs, siRNA targeting the HBV S gene. siHBx, siRNA targeting the HBV X gene. msiHBs, msiRNA targeting the HBV S gene. msiHBx, msiRNA targeting the HBV X gene

To investigate the knockdown effects of msiRNAs, various concentrations of msiHBs, msiHBx, siHBs, or siHBx were cotransfected with the HBV replication-competent plasmid pHY106-wta or pHY106-X15 into HepG2 cells. The ELISA results showed that the siRNAs knocked down the levels of HBsAg in the supernatants in a dose-dependent manner (Fig. 2). The levels of HBsAg decreased significantly even in the presence of very low concentrations (0.2 nM) of msiRNAs or siRNAs and to a level of less than 5% of untreated controls in the presence of 20 nM msiRNAs or siRNAs. Compared with the standard siRNAs (siHBs and siHBx), msiHBs was much less effective than siHBs, while msiHBx displayed a similar or even better inhibitory effect than siHBx on HBsAg production; therefore, msiHBx was used in subsequent experiments.

### msiHBx significantly inhibited the replication and gene expression of HBV in cells with transient or stable HBV replication

**Fig. 3:**
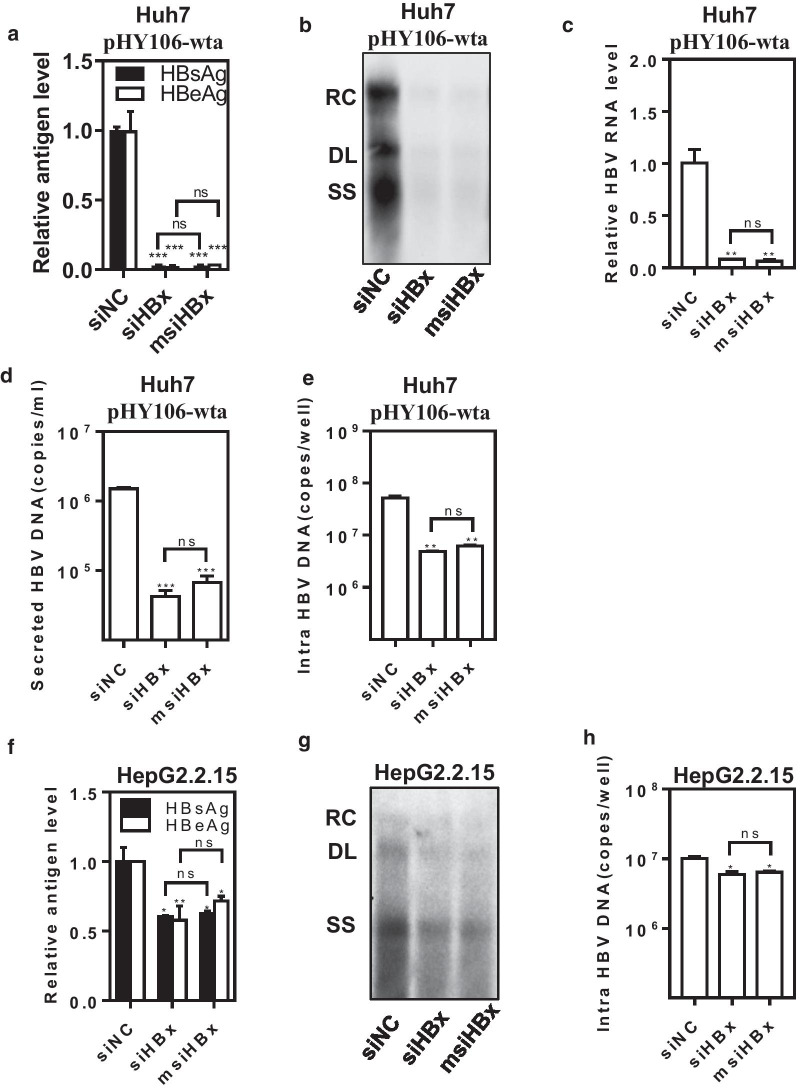
Inhibitory effects of msiHBx on HBV replication and gene expression. Huh7 cells were cotransfected with 1 µg of pHY106-wta and 20 nM msiHBx, siHBx, or siNC as a control. The levels of HBsAg and HBeAg in cell culture supernatants (**a**), HBV replicative intermediates (**b**, **e**), HBV DNA in cell culture supernatants (**d**), and HBV RNA in cells (**c**) were analyzed at 72 h after transfection by ELISA, Southern blotting, PCR, and One-Step RT-PCR, respectively. HepG2.2.15 cells were transfected with 20 nM msiHBx, siHBx, or siNC as a control. The HBsAg and HBeAg levels in cell culture supernatants (**f**) and HBV replicative intermediates (**g**, **h**) were analyzed at 72 h after transfection by ELISA, Southern blotting, and PCR, respectively. ns, no significant difference. *p < 0.05. **p < 0.01. ***p < 0.001. siNC was purchased from Sangon Biotech (Shanghai, China) and used as a negative control. siHBx, siRNA targeting the HBV X gene. msiHBx, msiRNA targeting the HBV X gene. RC, relaxed circular DNA. DL, double-stranded linear DNA. SS, single-stranded DNA

To further verify the inhibitory effects of msiHBx on HBV replication and gene expression, Huh7 cells were cotransfected with siHBx or msiHBx and the HBV replication-competent plasmid pHY106-wta. At 72 h after transfection, the levels of HBsAg and HBeAg in the supernatants (Fig. 3a), intracellular HBV RNA (Fig. 3c), and HBV DNA in the supernatants (Fig. 3d) decreased significantly to a level less than 5% of those treated with siRNA negative control (siNC). Compared with the siNC treatment, both siHBx and msiHBx treatment led to a significant decrease in HBV replication intermediates by over 90% (Fig. 3b, e). There was no significant difference between siHBx and msiHBx in the RNAi effects on HBV replication and gene expression (P > 0.05).

In HBV stably producing HepG2.2.15 cells transfected with siHBx or msiHBx, the levels of HBsAg and HBeAg in the supernatants (Fig. 3f) and intracellular HBV replication intermediates (Fig. 3h) were decreased to less than 60% compared with those in cells transfected with siNC. The reduction in HBV replication intermediates was also verified by Southern blotting (Fig. 3g). Again, there was no significant difference between siHBx and msiHBx in the inhibitory effects on HBV replication and gene expression in the HBV stably producing HepG2.2.15 cells (P > 0.05).

### msiHBx with a uridine-bulge modification enhanced antiviral cytokine secretion

**Fig. 4:**
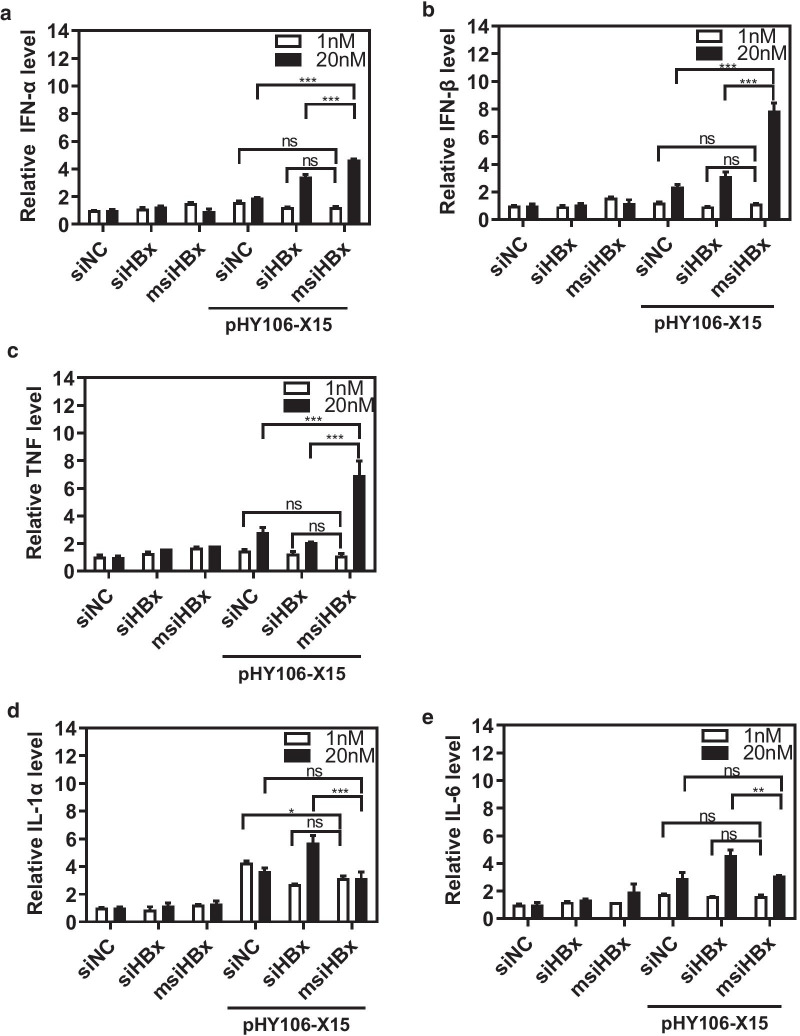
Enhanced antiviral cytokine secretion by msiHBx-directed HBV silencing. HepG2 cells were transfected with msiHBx, siHBx, or siNC alone or cotransfected with 1 μg of the HBV replication-competent plasmid pHY106-X15. The levels of IFN-α (**a**), IFN-β (**b**), TNF (**c**), IL-1α (**d**), and IL-6 (**e**) in supernatants were detected at 72 h after transfection by ELISA. ns, no significant difference. *p < 0.05. **p < 0.01. ***p < 0.001. siNC was purchased from Sangon Biotech (Shanghai, China) and used as a negative control. siHBx, siRNA targeting the HBV X gene. msiHBx, msiRNA targeting the HBV X gene

To explore whether msiHBx with an unpaired miRNA-like uridine-bulge modification could stimulate the innate immune response, HepG2 cells were transfected with 1 nM or 20 nM siHBx, msiHBx, or siNC alone as a control or in combination with the HBV replication-competent plasmid pHY106-X15. The results showed that in the absence of HBV, there was no significant difference in the levels of IFN-α, IFN-β, TNF-α, IL-1α, and IL-6 cytokine production in the supernatants of HepG2 cells treated with siHBx, msiHBx, or siNC. When msiHBx was cotransfected with the HBV replication-competent plasmid pHY106-X15 into HepG2 cells, a significant increase in the levels of the antiviral cytokines IFN-α (2.4-fold), IFN-β (3.3-fold), and TNF-α (2.4-fold) was induced compared with those in siNC-treated cells (Fig. 4a–c), but there was no significant increase in the levels of the inflammatory cytokines IL-1α and IL-6 (P > 0.05) (Fig. 4d, e). In contrast, in HepG2 cells cotransfected with siHBx and the HBV replication-competent plasmid pHY106-X15, a significant increase in the levels of IL-1α and IL-6 was observed (P < 0.01), and the levels of antiviral cytokines IFN-α and IFN-β increased to a lesser extent compared with those induced by msiHBx treatment.

**Fig. 5:**
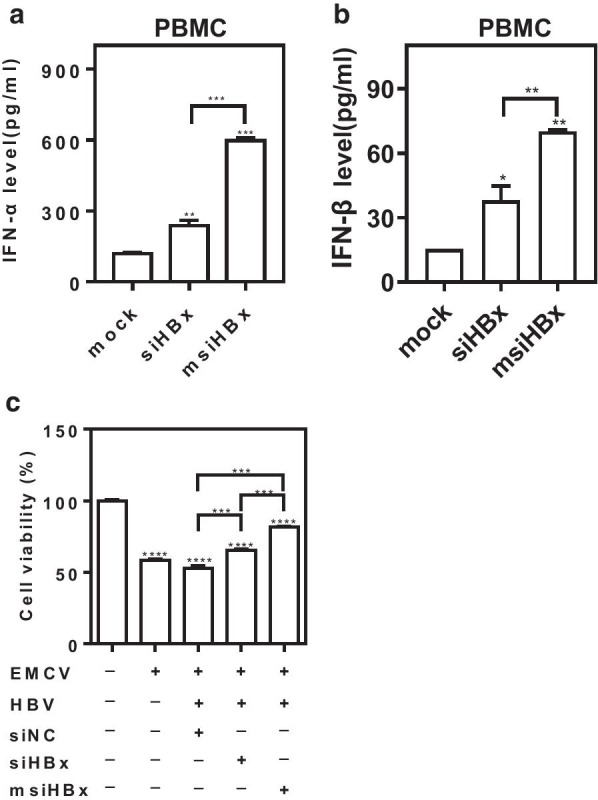
Type I interferons induced by msiHBx protected L929 cells from EMCV infection. PBMCs were transfected with siHBx or msiHBx at concentrations of 20 nM. The levels of IFN-α (**a**) and IFN-β (**b**) in supernatants were detected at 72 h by ELISA. The bioactivity of type I IFNs in cell culture supernatants from HepG2 cells cotransfected with 1 µg of pHY106-X15 and siHBx or msiHBx was determined by the virus protection assay (**c**). ns, no significant difference. *p < 0.05. **p < 0.01. ***p < 0.001. siNC was purchased from Sangon Biotech (Shanghai, China) and used as a negative control. siHBx, siRNA targeting the HBV X gene. msiHBx, msiRNA targeting the HBV X gene. PBMCs, peripheral blood mononuclear cells. EMCV, encephalomyocarditis virus

Further, when msiHBx or siHBx was transfected into PBMCs isolated from CHB patients, ELISA results showed that both msiHBx and siHBx caused a significant increase in the production of IFN-α and IFN-β, and msiHBx induced more IFN-α (fivefold *vs.* twofold, P < 0.01) and IFN-β (4.5-fold *vs.* 2.4-fold, P < 0.01) than siHBx (Fig. 5a, b).

### Type I interferons induced by msiHBx protected L929 cells from EMCV infection

In the virus protection assay, EMCV infection caused L929 cell death. However, in the presence of supernatants from HepG2 cells cotransfected with the HBV replication-competent plasmid pHY106-X15 and msiHBx or siHBx, the viability of L929 cells was significantly improved (P < 0.001), especially in the presence of supernatants from HepG2 cells cotransfected with pHY106-X15 and msiHBx (P < 0.001) (Fig. 5c), which indicated that type I interferons induced by siHBx- and msiHBx-directed RNAi protected L929 cells from EMCV infection.

## Discussion

In this study, in an HBV transient replication-competent system (HepG2 cells transfected with the HBV replication-competent plasmid pHY106-wta or pHY106-X15) and HBV stably producing HepG2.2.15 cells, the msiRNA msiHBx with a uridine-bulge modification significantly decreased the levels of HBV mRNA, the expression of HBsAg and HBeAg, HBV replication intermediates, and HBV virions. msiHBx showed similar or even better RNAi effects on HBV than the traditional siRNA siHBx targeting the same sequence, indicating that the RNAi activity of msiHBx was not affected by the uridine-bulge modification. On the other hand, msiHBx upregulated the expression of the antiviral cytokines IFN-α and IFN-β directly (msiHBx transfected into PBMCs) or indirectly by RNAi-directed immune stimulation (msiHBx cotransfected with the HBV replication-competent plasmid pHY106-wta or pHY106-X15 in HepG2 cells). The antiviral ability of type I interferons was confirmed in a viral protection assay, and msiHBx did not stimulate cells to secrete the inflammatory cytokines IL-1α or IL-6, suggesting that the msiRNA could activate cellular antiviral innate immunity without inducing an inflammatory response. In contrast, the HBV RNAi effects mediated by the traditional siRNA siHBx also caused the upregulated expression of the antiviral cytokines IFN-α and IFN-β, which was consistent with a previous study [13]. However, siHBx did not induce TNF-α but upregulated the inflammatory cytokines IL-1α and IL-6. In this study, compared with the traditional siHBx, msiHBx exhibited an enhanced ability to upregulate type I interferons without obvious inflammatory stimulation, which is beneficial for HBV immune control.

HBsAg and HBeAg play an important role in HBV-specific immune tolerance and persistent infection [6]. HBeAg loss/seroconversion is indicative of reduced virus replication, which is one of the goals of antiviral treatments. The consistent decrease in HBsAg facilitates the recovery of the HBV-specific immune response, which is beneficial for the immune control of HBV infection. “A functional cure”, which is characterized by HBsAg loss and/or HBsAg seroconversion, is the ideal treatment goal for HBV infection [15]. RNAi, with the ability to degrade mRNA in a sequence-specific manner, shows unique capabilities in reducing HBsAg and HBeAg [9,^ 22^, 23]. Preclinical and clinical studies have also confirmed that siRNA-based treatment could significantly reduce the levels of HBsAg and HBeAg in vivo and improve host immune function [24]. siRNA targeting the S gene (siHBs) can act on the 2.4-kb and 2.1-kb HBV mRNA to reduce HBsAg, preS2, and preS1 protein levels and act on the 3.5-kb HBV pregenomic RNA (pgRNA), leading to the degradation of HBV pgRNA and reduction in HBcAg and HBeAg, altogether resulting in the inhibition of HBV replication and gene expression. siRNA targeting the X gene (siHBx) can act on all HBV mRNAs. Therefore, in addition to the above effects, siHBx also degrades the 0.7-kb HBx mRNA, thereby downregulating the expression of HBxAg. Since HBxAg plays an important role in maintaining the stability of HBV covalently closed circular DNA (cccDNA), inhibition or mutation of HBxAg can decrease the level of cccDNA [25]. Therefore, siHBx can theoretically exert a better anti-HBV effect than siHBs; thus, the siHBx targeting the X gene was selected for further investigation in this study.

The presence of uridine in RNA induces TLR7/8-dependent cytokine production, which correlates with overall uridine moieties [13,^ 26^, 27]. The insertion of unpaired miRNA-like uracil at the 9–12 bases of the siRNA messenger strand will enhance the immunostimulatory ability of the siRNA as the “U” content of the siRNA increases. On the other hand, the miRNA-like stem-loop structure of msiRNA can also enhance the immunostimulatory activity of an siRNA through TLR7/8 [28]. Michael et al. [28] designed a bifunctional siRNA with both immunostimulatory and specific silencing activities. The introduction of a miRNA-like unpaired uridine-bulge modification strongly stimulated human immune cells, leading to the production of cytokines, thereby protecting HeLa cells from Semliki Forest virus infection.

In this study, the RNAi activity of siHBs was significantly affected after the introduction of unpaired uridine-bulge (msiHBs), while the RNAi activity of msiHBx was consistent with or even better than that of siHBx. A deeper look into the secondary structure of the two msiRNAs indicates that the 4 “U” of the 9–12 bases of msiHBs are unpaired, while only 2/4 “U” of the 9–12 bases of msiHBx are unpaired. Thus, the larger ring-shaped protrusion of msiHBs may affect the binding efficiency or interaction of msiHBs and Dicer, thereby accounting for the reduced RNAi activity.

A significantly increased production of IFN-α and IFN-β was induced in PBMCs transfected with msiHBx, further validating the immunostimulatory activity of msiHBx. No obvious cytokines were found to be upregulated in HepG2 cells transfected with msiHBx or siHBx. This is due to defects in the innate immune signaling pathways of hepatocarcinoma cells, which require stronger stimulation signals to activate [6]. Transfection of the traditional siRNA siHBx into PBMCs also induced IFN-α and IFN-β, suggesting that siHBx itself also possesses certain immunostimulatory activity, but siHBx-mediated IFN-α and IFN-β production was much lower than that induced by msiHBx, further confirming that a uracil ring-shaped protrusion modification can enhance the immunostimulatory activity of siRNAs. RNAi-mediated degradation of target mRNA can activate cellular innate immunity through the TLR-7/8 or PKR pathway [6]. In this study, both msiHBx- and siHBx-directed HBV RNAi (msiHBx or siHBx cotransfected with pHY106-wta in HepG2 cells) upregulated the expression of IFN-α and IFN-β, providing more evidence in favor of RNAi-mediated immune stimulation. Compared with siHBx, msiHBx-directed HBV RNAi induced higher levels of IFN-α and IFN-β, further indicating that the presence of the uracil ring-shaped protrusion structure can enhance the immunostimulatory activity of RNAi. Interestingly, HBV RNAi directed by the traditional siRNA siHBx stimulated the cells to produce the inflammatory cytokines IL-1α and IL-6, while HBV RNAi directed by the msiRNA msiHBx did not induce IL-1α or IL-6. msiHBx can enhance the antiviral innate immunity of cells without activating the cellular inflammatory response. This characteristic of msiRNAs is more conducive to the immune control of HBV and limits immune-related inflammatory damage. Therefore, msiRNAs can enhance the secretion of antiviral factors without activating the cellular inflammatory response, in addition to inhibiting the replication and gene expression of HBV. This characteristic of msiRNAs may play an important role in the immune control of the virus and may be developed as a new anti-HBV agent.

## Conclusions

In Conclusion, high levels of HBV virions and proteins are key factors for maintaining persistent infection. Effective anti-HBV treatment strategies need to reduce the HBV loads and antigens on the one hand and restore/enhance the anti-HBV immune response on the other hand. Here, the msiRNA modified by the uracil ring-shaped protrusion structure enhanced the immunostimulatory activity of RNAi without inducing cell inflammation beyond the high RNAi activity. The antiviral/immunostimulatory dual activities of msiRNA are more conducive to the control of HBV, and it is unnecessary to consider immune-related inflammatory damage to the liver. The in vivo antiviral and immunostimulatory activities of the msiRNA and the role of immunostimulatory activity in immune control and clearance of HBV deserve further study.

## Data Availability

The datasets used and analyzed during the current study are available from the corresponding author on reasonable request.

## Abbreviations

CHB: Chronic hepatitis B
HBV: Hepatitis B virus
PEG-IFN-α: Pegylated interferon-α
NAs: Nucleos(t)ide analogs
TAF: Tenofovir alafenamide fumarate
HBsAg: Hepatitis B surface antigen
HBeAg: Hepatitis B e antigen
TLR: Toll-like receptor
NPCs: Nonparenchymal liver cells
RNAi: RNA interference
siRNA: Small interfering RNA
RIG-I: Retinoic acid-inducible gene-I
MDA5: Melanoma differentiation-associated protein 5
PKR: Protein kinase receptor
isiRNAs: Immunostimulatory siRNAs
GP: Guanidinopropyl
IL: Interleukin
TNF: Tumor necrosis factor
3p: Triphosphate
miRNA: MicroRNA
msiRNAs: MiRNA-like siRNAs
FBS: Fetal bovine serum
NEAA: Nonessential amino acids
PBMCs: Peripheral blood mononuclear cells
RT: Reverse transcription
EMCV: Encephalomyocarditis virus
pgRNA: Pregenomic RNA
cccDNA: Covalently closed circularDNA
